# Variation in veterans affairs hospitals’ UV-C surface disinfection systems guidance documents: a qualitative content analysis

**DOI:** 10.1017/ash.2025.10047

**Published:** 2025-06-20

**Authors:** Julia Friberg Walhof, AM Racila, Stacy Hockett Sherlock, Cassie Cunningham Goedken, Daniel Suh, Bernardino Guerrero, Trina Zabarsky, Kimberly Dukes, Eli N Perencevich

**Affiliations:** 1 Center for Access and Delivery Research and Evaluation, Iowa City Veterans Affairs Health Care System, Iowa City, IA, USA; 2 Division of General Internal Medicine, Department of Internal Medicine, Carver College of Medicine, University of Iowa, Iowa City, IA, USA; 3 Environmental Programs Service (EPS), Veterans Affairs Central Office, Washington, DC, USA

## Abstract

The Department of Veterans Affairs (VA) does not have system-wide standardized policies or procedures for ultraviolet-C (UV-C) use. Qualitative researchers performed content analysis of VAV UV-C guidance documents. We observed that lack of specificity and uniformity across guidance documents is a potential barrier to UV-C implementation and future quality control.

## Background

Evidence suggests ultraviolet-C (UV-C) surface disinfection systems technology can be useful for reducing a variety of potentially harmful pathogens on surfaces and preventing healthcare-associated infections,^1^ when added to standard manual cleaning and disinfection practices.^2–5^ Effectiveness of UV-C varies depending on the device used and the manual cleaning protocol.^6^ Interest in UV-C disinfection has increased since the COVID-19 pandemic.^2^ However, lack of information and standardization around current UV-C policies persists, contributing to reduced efficacy and cost-effectiveness.^4,7^ The Department of Veterans Affairs (VA) does not have system-wide standardized policies or procedures for UV-C use. We sought to better understand if and how individual VA health system facilities describe recommended use of UV-C technology. Understanding variation in UV-C guidance documents within VA could help optimize UV-C implementation.

## Methods

As part of a larger mixed-methods implementation effectiveness study, two qualitative researchers performed a combined inductive-deductive approach to content analysis^8^ of VA documents guiding UV-C policy and operations. Between June and August 2023, researchers solicited additional UV-C documents to supplement documents previously collected during nationwide interviews with multidrug-resistant organism (MDRO) coordinators and environmental management service (EMS) staff. Researchers also emailed additional MDRO and EMS VA staff who hadn’t been interviewed, asking for “policies, standard operating procedures (SOPs), and/or guidance related to UV-C use in your institution.” Researchers logged email responses and any documents received, then performed content analysis of all documents using an Excel spreadsheet matrix. Lead coders sorted data into thematic and topical categories of interest. Categories were summarized and reviewed along with emergent findings by the study team. Lead coders developed a coding system, brought the system to a larger group for discussion and validation, and shared any unresolved differences with the principal investigator for final determination.

## Results

**Figure 1.**
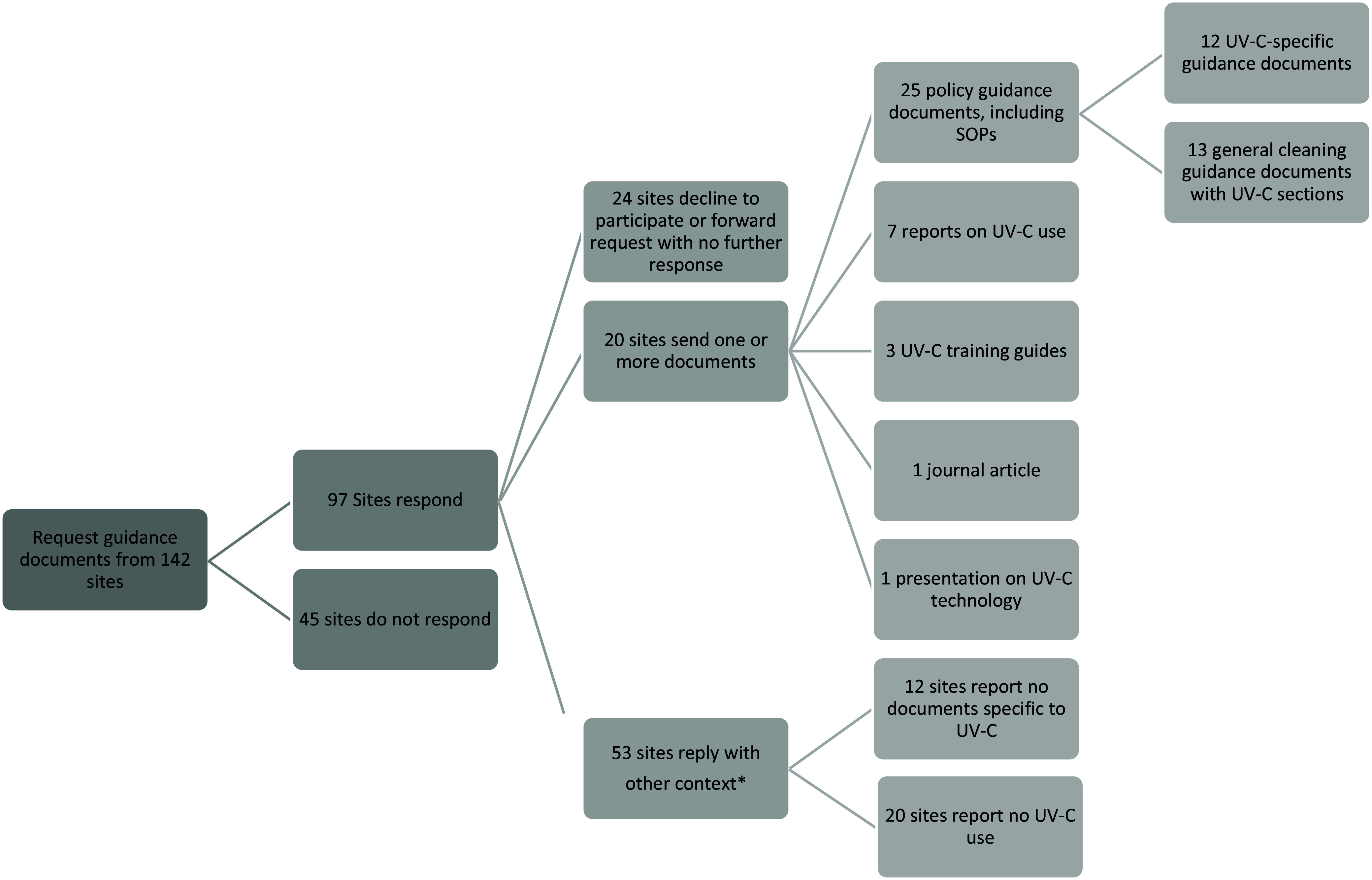
Breakdown of response information and type of policy guidance documents received from sites. * Context included information participants sent us in email correspondence about whether and how UV-C technologies were used in their facilities. This information was sent to us in the body of the emails, outside of guidance documents.

Researchers received responses from VA facilities across the country. In total, 73 of 142 (51%) facilities responded with context about their UV-C use. We received guidance documents from 20 of the 73 responding sites, and another 20 reported no UV-C use. Most materials received were documents tailored for guiding manual cleaning and disinfection in VA Medical Centers (Figure 1). UV-C was often included within guidance documents for more general environmental cleaning and disinfection, rather than having its own separate guidance document. Guidance documents mentioned UV-C use as part of various cleaning routines, including use in ORs and EDs (n = 15), terminal cleaning in patient rooms (n = 7), and disinfection after the last procedure of the day (n = 6), in addition to two-step manual cleaning and disinfection of high-touch surfaces and objects in non-inpatient rooms like ORs and EDs (n = 7). Some facilities also specified UV-C use in cardiac catheterization laboratories (n = 7) or for small handheld items (n = 1) or vehicles (n = 1) but not larger rooms. All guidance materials indicated UV-C use following patients with contact precautions or COVID-19.

Eleven facilities that replied with current context of UV-C use lacked specified guidance documents for UV-C. One facility reported they developed an SOP following our inquiry. Another facility was in the process of creating an SOP for UV-C disinfection use during all isolation precaution room discharges. Some facilities reported they relied on manufacturers’ literature to guide use in place of SOPs or policies, or to help guide SOP or policy development.

When described in documents received, UV-C cycles lasted on average of 5 minutes, and some rooms required multiple cycles. When described, wavelength was defined as “full-spectrum UV light.” Portable disinfection “robot” devices were mentioned most often. Handheld and overhead UV-C units were less commonly used. We found UV-C operation most often fell under the purview of EMS. One facility reported that maintenance of their overhead UV-C lighting system fell under their engineering department. Some facilities also occasionally consulted their infection prevention and control programs about UV-C operation effectiveness.

Norovirus, COVID-19, and *Clostridioides difficile* were the most common pathogens mentioned in documents. Guidance documents were not always updated in a timely manner, for instance to reflect the use of UV-C against COVID-19. Fourteen of the documents were published between 2017 and 2020, and 11 of the documents were published between 2021 and 2023.

Documentation rarely specified personal protective equipment use with UV-C. Gloves and UV-C-appropriate eye protection were mentioned when specified. General safety guidance when described included strategies such as ensuring the room is empty, setting up caution signs and physical barriers to the room such as cones or barrier chains, closing doors into the room, and curtains of room windows, and setting up UV-C machine deactivation sensors. Restarting the machine was the most common guidance offered for addressing UV-C malfunction, although this was rarely specified.

## Discussion

When implementing new policies, programs, and/or guidance at a local level, it is important to consider the local context. Demeersseman et al. and Periera et al. described a general lack of standardization and information about policies and guidance for UV-C use, potentially limiting effectiveness to the detriment of reducing the risk of infection for patients and cost-effectiveness of expensive equipment.^4,7^ The VA responses and documents we received also lacked uniformity and standardization in guiding the use of VA UV-C systems. There was variability with how UV-C technology was incorporated into SOP, policy, and/or other guidance documents. Furthermore, we seldom received UV-C-specific SOPs from the VAs we queried. Often, UV-C was not a separate item but included within documents guiding overall environmental cleaning procedures. This lack of specificity and uniformity is a potential barrier to UV-C implementation, with potential to hinder future quality control. Guidance variability may also explain some of the wide range of UV-C benefits when these systems were implemented in a large proportion of VA sites.^1^ Our findings highlight that UV-C-specific guidance documents are not standardized across the VA system, and there is a need for developing standardized national policies, guidelines, and/or other guidance documents, which can then be tailored to the local facility’s context. Additionally, since many guidance documents may be outdated, standardized guidance documents should also be periodically reviewed to ensure they remain up-to-date with facility equipment, standards, and pathogens to help improve overall effectiveness.

Limitations of this analysis include the reliance on guidance documents, which may not fully reflect current practice. There is also the potential for possible bias because not everyone we reached out to replied to our requests for information, and those that did respond were self-reporting their guidance document information. However, this report provides a first step to understanding how UV-C technology is used in the VA and, with further research, how it can be more effectively employed in hospital disinfection and sanitization practice.
